# Texas Medical Association

**Published:** 1894-10

**Authors:** 


### EDITORIAL DEPARTMENT.

F. E. DANIEL, M. D., Editor.
S. E. HUDSON, M. D., Managing Editor.
A. J. SMITH. M. D., Galveston, Associate Editor.

EDITORIAL STAFF:

PROF. J. E. THOMPSON, M. D., Texas Medical College, Galveston; Surgery.
PROF. WM. KEILLER, M. D., Texas Medical College, Galveston; Obstetrics and Gynecology.
PROF. DAVID CERNA, M. D., Texas Medical College, Galveston; Therapeutics.
PROF. A. J. SMITH, M. D., Texas Medical College, Galveston; Medicine
DR. ISADORE DYER, Tulane University; Dermatology.
DR. R. H. L. BIBB, Saltillo, Mexico; Foreign Correspondent.
DR. ROBT. MORRIS, Charity Hospital, N. O.; Clinical Reports

### TEXAS STATE MEDICAL ASSOCIATION.

In our last issue we advocated changing tactics in the undertaking to organize the medical profession of Texas. We showed that in twelve years, dating from April, 1882, there had been, up to and including the meeting of 1893, a net gain of only 106 members, or less than nine members each year. This is, indeed, hastening slowly, and is very discouraging to those who have worked hard for the cause, and whose sincere wish it is to see the entire regular profession of the State enrolled under the banner of rational medicine, and organized for effective work. The result is still more disheartening when it is remembered that the Association was old,—quite mature,—when our observation begun, in 1882; that it was organized as far back as 1869, "for the purpose of unifying the profession of Texas and the advancement of medicine," and had been pegging away, with indifferent success, ever since, there never having been on the roll, at any one time, more than about five hundred members. We pointed out how that, at each meeting, large accessions to the membership are made, and the reasons why they do not stick,—the non-payment of dues, principally, caused by inability to attend the meetings consecutive years. We advised abandoning the plan upon which we have been working all these years to no purpose, and suggested that in lieu of a State Medical Association by membership, the energies of leading physicians be directed to organizing each county or district, and having every local society send representatives, once a year, to Austin, to a grand State Medical Convention.

In looking over the Transactions for 1894, just issued, we are still further impressed with the belief that we can never succeed in holding together more than six or eight per cent. of the profession under the present plan, for there has been a still further falling off in numbers, and consequently in receipts, and the treasury is now empty.

Secretary West, in his last report, says:

MEMBERSHIP.

| | | |
|---|---|---|
| There were on the roll May 1, 1893 .................. | | 406 |
| Added—names dropped by mistake.................... | | 2 |
| New members elected at Galveston.................... | | 37 |
| Total [honorary members omitted.—Ed.] ....... | | 445 |
| Dropped for non-payment of dues ..................... | 42 | |
| Resigned ........................................ | 3 | |
| Died [active members, not ex-members.—Ed.] ........ | 2 | 47 |
| Showing a balance on the roll at this meeting of....... | | 398 |

But by actual count there are on the rolls exactly 354, including those admitted at Austin in April last, about 35. This shows a discrepancy between the rolls and the Secretary's report of 44 members, who, it is supposed, must have been dropped for non-payment of dues since the April term.

It is thus seen that there are now only 74 members more than there were in 1882 (we had then 280), a net gain of less than six in a year. Comment is unnecessary. A change of tactics is imperative, if we ever expect to have a *State* Medical Association, for as it stands it is not,—in fact, never has been,—a "State" Medical Association, in that the whole State is not represented by membership.

A WORD ABOUT THE TRANSACTIONS.

In light of the foregoing facts, and in further consideration of the remarkable business stagnation and depression,—the terrible tight times,—it was a grave error of judgment in our ambitious young Secretary to get out the Transactions in such expensive style. Of all the years of the Association's existence, this is the worst one in which he could have bankrupted the treasury by a costly volume of Transactions, thereby entailing an additional tax upon the members to make good the deficit. It is surprising, too, because Dr. West foresaw the necessity of economy. In his last report, in presenting the beautiful volume of Transactions of 1893, he said:

"At the outset of publication, it became a question whether the Treasurer would have the necessary funds to pay the bill, and it was thought we would have to levy a small assessment. Fortunately we did not have to do this, but last year's experience forces upon up the question of economy. I would suggest as one means to this end, that authors, in future, be required to pay for their own illustrations. If the volume of this year should equal or exceed in cost that of last year, we may have to consider the question of leaving off the cloth binding, by which means about seven per cent. of the total cost might be saved. These questions are presented for your consideration now, as action may be demanded upon them very soon. While it is incumbent upon us to maintain a high standard of excellence, both in appearance and quality, of our annual volume of Transactions, at the same time it is absolutely necessary to keep expenses within the bounds of our receipts."

And then he deliberately transcended the bounds, and, we learn from President McLaughlin, will have to "levy a small assessment" at Dallas.

Nor was it necessary to spend so much money for the Transactions, even had the Association been flourishing, instead of falling off in numbers, and had plenty money, instead of being as he pointed out, threatened with bankruptcy. "A high standard of excellence" in the get up of our Transactions, Dr. West's justification and plea, had been established long before Dr. West's election; as far back as 1885, when Burt was Secretary. The style (purple cloth binding), inaugurated under Burt's administration in 1885, was continued by his successor in office, Dr. Daniel, and the volumes of 1886, 1888, 1889 and 1890 were pronounced by leading medical journals, "*the handsomest and most creditable volume of yearly transactions issued by any State Medical Association*," and these volumes cost less than half the cost of either of the last two issued by Dr. West. Why not be satisfied with a volume pronounced "better than the best"? Why improve on it?

But it is not so much the "improvement"; because those of 1885, 1886, '88, '89, '90 will compare in appearance most favorably with those issued since. Secretary West paid more for his work than it had been done for at Austin, and he employed finer paper and smaller type; there is more matter to the page, and in the last two volumes, there are more pages to the volume than any preceding volume, except 1886.*

Dr. West says, the volume of 1893—448 pages—cost $1.80 per volume delivered. As 1894 is just like it, and has 450 pages, it is to be supposed it did not cost less.

The volume of 1889—340 pages—cost 78 cents per volume, or 90 cents delivered, just one-half the cost of 1893 and 1894; the volume of 1890—342 pages—cost 84 cents and 12 cents for postage, making 96 cents delivered; and we contend these were good enough, and the members were abundantly satisfied. We claim, too, that the work could have been done for less, and the Secretary is not to be held blameless for exceeding the resources of the treasury under the circumstances. His predecessors advertised for bids, and got the best work for the least money. And that plan should have been followed; but to our certain knowledge, Secretary West refused to let the Austin printers bid who had done the work for the several years previous to 1891, and at such reasonable figures and so satisfactorily. The members—the few who have remained faithful—are in no mood to submit to further taxation, and indeed few of them are able to stand a raise; many are grumbling at the $5 membership fee, and some have dropped out solely on that account, saying, if they can not attend the meetings they get no benefit *except* the Transactions, and they think $5 quite enough for that luxury.

The above is written in no spirit of captiousness, but from a sense of duty. The JOURNAL has, all along, been devoted to the Association's best interests, and it enters its protest against such extravagance. The Transactions could and should be gotten out in less expensive style, especially when there is no money to pay for it.

---

### PENNY WISE AND POUND FOOLISH.

Apropos of our editorial on the extravagance of the Publishing

---

*The volume of Transactions that year contained nearly 800 pages. The publishing committee objected to publishing certain matter, for one reason —they knew there was not money enough to pay for it, but were compelled to publish it all, by order of the then President, Nott. 750 volumes were published—the Association then numbering over 500 members. The total cost was $1185.00, or $250 more than was in the treasury. This amount the committee advanced out of their own pocket, and were reimbursed next meeting. Notwithstanding the 792 pages, the book cost only $1.55 per copy. As a result, next year's volume, 1887, had to be issued in paper covers.—ED.

Committee in bankrupting the treasury in getting out the Transactions, we mention that a beautiful half-tone engraving of President McLaughlin, made for, and published in the TEXAS MEDICAL JOURNAL for May, was offered Secretary West, free of cost, to be used in the Transactions. His predecessor had introduced the practice of publishing the President's picture every year in the Transactions, and with the exception of Dr. Sears and Dr. Wilkes, the JOURNAL published the picture of every President since 1886, turning the cut over then to the Publishing Committee for use in Transactions, without cost to the Association. Secretary West declined to publish Dr. McLaughlin's picture, because of the additional expense he said it would entail. In this connection we will remark, that authors of papers requiring illustration, were required to pay for the cuts, and in one instance brought to our notice—the author was asked *to pay for printing* the cut illustrating his paper (Dr. M. M. Smith). We feel quite satisfied that if called upon to do so, Dr. McLaughlin, our handsome President, the cut having been paid for, would cheerfully have paid the two or three dollars the printing and binding would have cost. We are not advised as to the extent of the deficiency; but the printing of the President's picture—which would have made the volume *complete*—would not have added greatly to it.

The JOURNAL'S warning some months ago—"Retrench or Bankrupt"—was not only not heeded, but was resented as a piece of impertinence. We trust the animus of this may not also be misunderstood. For the Secretary we have the kindest of feelings; but we can not endorse his action in the premises,—an error of judgment, no doubt, but still an error and a serious one.

