# The Transactions: A Correction

**Published:** 1894-11

**Authors:** J. W. McLaughlin

**Affiliations:** President Texas State Medical Association; Austin, Texas


### The Transactions: A Correction.

AUSTIN, TEXAS, October, 1894.

*Editor Texas Medical Journal:*

Your editorial in the October issue of the JOURNAL, headed "Penny Wise and Pound Foolish," is not correct in saying that Dr. West, Secretary of the State Medical Association, declined to insert the President's picture in the last volume of Transactions, on the plea of economy, notwithstanding the engraving was offered to him free of cost. Believing, as I do, that this statement is based upon a misapprehension of the facts, and that you would not knowingly do Dr. West, or any one else, an injustice, I respectfully request that you publish this communication in the November issue of the JOURNAL, in order that the matter may be correctly understood.

I am free to admit that it would have given me pleasure to have had my picture and biographical sketch appear in the beautiful volume of Transactions which Dr. West has given us, but in view of the probable deficit that confronts us, and my desire to not increase this by an unnecessary expenditure of Association funds, I thought it wise to limit the volume to the strictly legitimate work of the Association, and therefore declined Dr. West's request that I furnish him with the engraving and biographical sketch for the Transactions.

Very respectfully,

J. W. McLaughlin, M. D.,
President Texas State Medical Association.

